# OCT angiography features associated with macular edema recurrence after intravitreal bevacizumab treatment in branch retinal vein occlusion

**DOI:** 10.1038/s41598-019-50637-8

**Published:** 2019-10-02

**Authors:** Kwang-Eon Choi, Cheolmin Yun, Jaehyung Cha, Seong-Woo Kim

**Affiliations:** 1 Department of Ophthalmology, Korea University College of Medicine, Seoul, South Korea; 2 Medical Science Research Center, Korea University College of Medicine, Seoul, South Korea

**Keywords:** Retinal diseases, Eye manifestations

## Abstract

We aimed to evaluate the relationship between the capillary abnormalities including nonperfusion area (NPA) in optical coherence tomography angiography (OCTA) images and the recurrence of macular edema (ME) secondary to branch retinal vein occlusion (BRVO) after intravitreal injection of anti-vascular endothelial growth factor (anti-VEGF; bevacizumab). The records of 40 patients who underwent intravitreal bevacizumab injection for ME secondary to BRVO and had at least six months of follow-up were reviewed. Central retinal thickness (CRT; μm) and macular edema type were evaluated prior to treatment. After ME resolution, nonperfusion areas in the 1 mm (NPA1) and 1–3 mm (NPA3) zones on the Early Treatment Diabetic Retinopathy Study (ETDRS) circle within the superficial capillary plexus (SCP) and deep capillary plexus (DCP) were measured using OCTA images. Furthermore, other microvascular abnormalities in the both SCP and DCP were compared between groups. ME recurred in 25 of 40 (62.5%) eyes. The NPA1 of the SCP and DCP (p = 0.002, 0.004, respectively), NPA3 of the SCP and DCP (p = 0.002, 0.008, respectively), and initial CRT (p = 0.022) differed significantly between eyes with and without ME recurrence. In multivariate logistic regression analyses, the NPA1 of the DCP (OR: 344.718; p = 0.029) and NPA3 of the SCP (OR: 4.072; p = 0.018) were significantly associated with ME recurrence. Other microvascular abnormalities were not significantly different between two groups. The central NPA and parafoveal NPA of the SCP in OCTA images correlated strongly with ME recurrence in BRVO patients after intravitreal anti-VEGF injection.

## Introduction

A branch retinal vein occlusion occurs when branches of the retinal venous drainage system become blocked. The prognosis and visual outcome depend on the amount of retinal ischemia^1^. Macular edema (ME) with BRVO is an important cause of impaired vision^2,3^. ME may be due to a leakage of fluid from capillaries that are upstream of or proximal to the obstructed vein^4^. BRVO and related ME resolve spontaneously within one year in about 50% of patients, but persistent or relapsing ME can often hinder the recovery of visual acuity^5^. BRVO has been classified as perfused (nonischemic) and nonperfused (ischemic). Ischemic BRVO is defined as >5 disc diameters of nonperfusion on fluorescein angiography (FA)^6,7^. According to one study, the visual prognosis in eyes with BRVO showing ischemic ME is better than in those with nonischemic ME^8^. In contrast, patients with central retinal vein occlusion (CRVO) who have better retinal perfusion and less retinal ischemia have better visual outcomes after aflibercept injection^9^. Vascular endothelial growth factor (VEGF) plays a key role in the pathogenesis of ME secondary to BRVO^10^. The severe ME and concomitant capillary ischemia present in BRVO can be associated with high levels of intraocular VEGF^11^. ME following BRVO can be treated with macular grid laser photocoagulation, intravitreal anti-VEGF injections, corticosteroids, and so on. Intravitreal anti-VEGF injection is the gold standard first-line treatment for ME^12,13^. However, some patients show persistent or relapsing ME, even after repeated treatment with anti-VEGF agents^14^. In one study using FA images, the degree of ME or extent of the nonperfusion area (NPA) was correlated with visual prognosis and response to anti-VEGF therapy^15^.

With regard to the recurrence of ME in BRVO cases, our previous study of FA images showed that the degree of macular ischemia was associated with ME recurrence after intravitreal anti-VEGF injection^16^. Overall retinal vascular changes, such as macular capillary NPAs in BRVO, have been visualized by FA^17^, but specific evaluation of the vasculature at each capillary level is not possible with FA. Several studies in humans and animal models have shown that major arterioles and especially major venules can independently connect to the deep capillary plexus (DCP) without first communicating with the superficial capillary plexus (SCP) and also that the DCP could have a primarily venous role^18–24^. A recently developed optical coherence tomography angiography (OCTA) technique can noninvasively visualize and differentiate the SCP and DCP, allowing for analyses of the microvascular abnormalities of each retinal capillary plexus^25,26^. Some reports in which OCTA was performed in eyes with BRVO have concluded that retinal vascular changes, such as NPAs and microaneurysms, develop more frequently in the DCP than in the SCP^27–29^.

In the present study, we measured the vascular NPA of each capillary plexus using OCTA images of BRVO patients with ME. We also evaluated the ME type in optical coherence tomography (OCT) images before treatment and microvascular abnormalities in OCTA images after ME resolution. We evaluated which OCTA features are associated with ME recurrence after anti-VEGF treatment.

## Materials and Methods

### Patients

The institutional review board (IRB) of Korea University Ansan Hospital approved this study, waiving the requirement for informed consent for study participation. All research protocols and methods of data collection adhered to the tenets of the Declaration of Helsinki.

This retrospective study included patients who underwent off-label intravitreal anti-VEGF (Avasin^®^, bevacizumab; 1.25 mg in 0.05 ml) injection for ME secondary to unilateral BRVO at Korea University Ansan Hospital between January 2016 and March 2018. The study inclusion criteria were as follows: (1) unilateral BRVO onset within three months; (2) baseline examinations performed within the two-week period preceding the initial intravitreal anti-VEGF injection; (3) ME resolution after the first or second intravitreal anti-VEGF injection; (4) OCTA imaging at one or two months after ME resolution showing no major blocked signals associated with retinal hemorrhage; (5) OCT images with a signal-to-noise ratio of 0.6 or greater; and (6) a central retinal thickness (CRT) of ≥300 μm as measured by OCT at the first evaluation. The exclusion criteria were as follows: (1) a follow-up period of less than six months after intravitreal anti-VEGF injection; (2) the presence of other retinal disease, such as diabetic retinopathy or epiretinal membrane; (3) a history of other intraocular treatments, such as intravitreal triamcinolone injection or focal/grid laser photocoagulation; (4) CRVO or hemi-CRVO.

All patients underwent complete ophthalmic examinations prior to injection, including best-corrected visual acuity (BCVA) measurements (expressed as log MAR units), slit-lamp examination, dilated fundus examination, ultra-wide-field color fundus photography (WFP), FA, and OCT at the first visit (baseline assessment). All follow-up examinations, including OCT imaging, were repeated at intervals of 2–3 months after the 1-month follow-up visit. All patients received one initial intravitreal injection (IVR). And patients with persistent ME at a subsequent follow-up visit, received an additional intravitreal anti-VEGF injection^30^. If bevacizumab was injected again, the follow-up schedule like after 1st injection was reimplemented. BCVA measurement, slit-lamp examination, dilated fundus examination, WFP, OCT, and OCTA were performed one month after the initial intravitreal anti-VEGF injection. The development of ME was defined as a CRT ≥300 μm. Resolution of ME was defined as a CRT <300 μm after intravitreal anti-VEGF injection. Likewise, the recurrence of ME was defined as a CRT ≥300 μm after the resolution of ME or a CRT of <300 μm with a new retinal cyst or subretinal fluid development within six months of one or two intravitreal anti-VEGF injections.

### Optical coherence tomography and optical coherence tomography angiography

OCT and OCTA images were obtained using a Spectralis OCT imaging device (version SP 6.7.21.0, 870 nm wavelength; Heidelberg Engineering, Heidelberg, Germany) at Ansan Hospital. All of the OCTA examinations were performed using a 3.0 mm by 3.0 mm volume scan pattern centered on the fovea according to the analysis protocol (FS-ADA) and variables.

### Image analysis

The types of ME in the initial OCT images were evaluated and classified as follows^16^. Serous retinal detachment was defined as retinal thickening with subretinal fluid, and cystoid ME was defined as retinal thickening with intraretinal cystic areas of low reflectivity. The third type was mixed serous retinal detachment and cystoid ME, and the fourth type was sponge-like ME, defined as diffuse retinal thickening with retinal layers having a sponge-like appearance.

**Figure 1:**
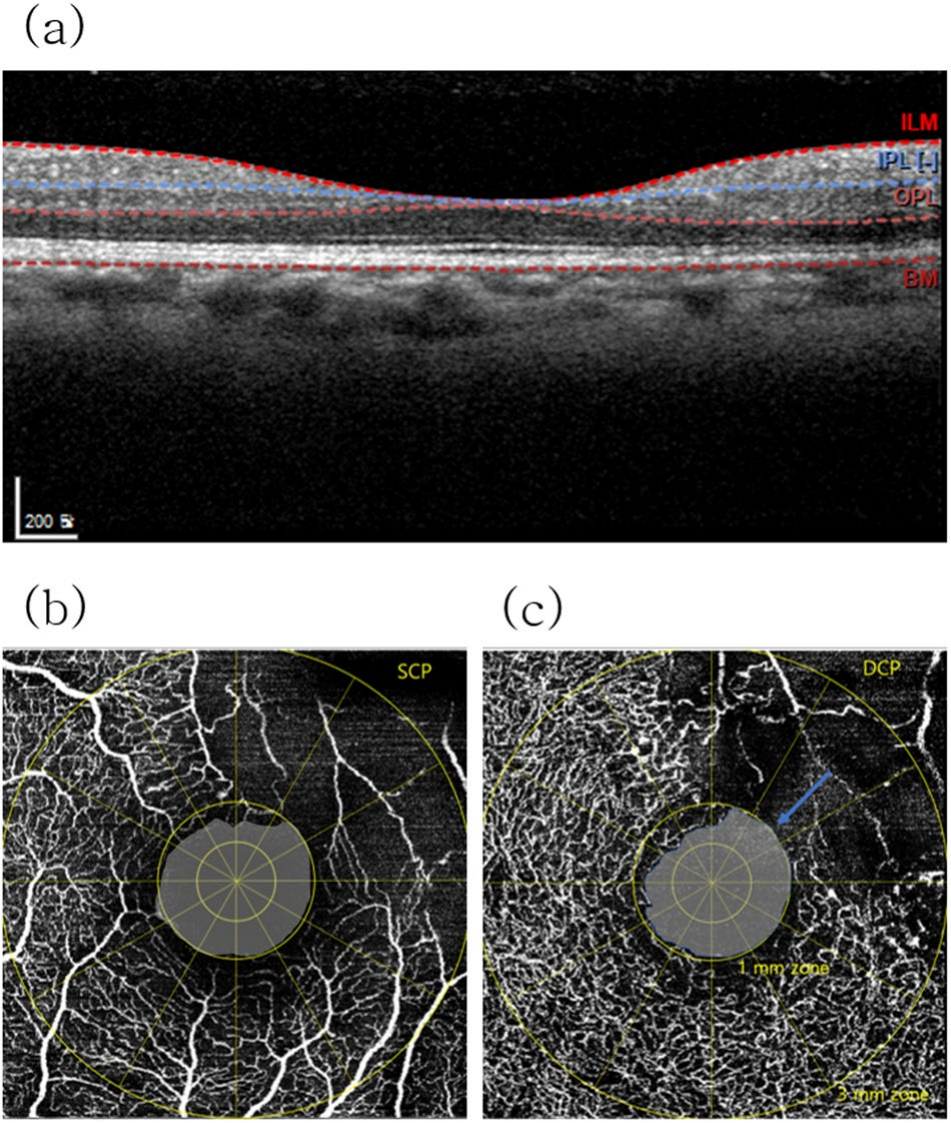
(**a**) Optical coherence tomography image and (**b**,**c**) Optical coherence tomography angiography (OCTA) images obtained using the Spectralis OCTA device (Heidelberg Engineering, Heidelberg, Germany). (**b**,**c**) In this case, the superior border of the foveal avascular zone (FAZ) was vague due to vascular dropout (blue arrow). Nonperfusion area 1 (NPA1) of (**b**) the superficial capillary plexus (SCP) and (**c**) the deep capillary plexus (DCP) was defined as the dropout-area in the 1-mm zone. The NPA1 of each capillary plexus was delineated as the gray area in the 1-mm zone.

**Figure 2:**
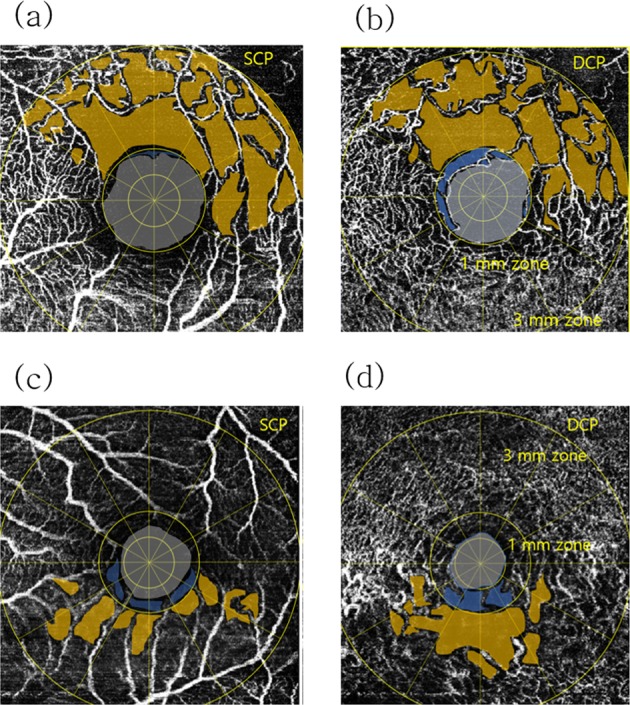
Schematic nonperfusion area (NPA) images from (**a**,**b**) a 62-year-old female patient from the recurrence group and (**c**,**d**) a 61-year-old female patient from the nonrecurrence group. Nonperfusion area 3 (NPA3) was defined as the capillary dropout area plus the dark or gray areas with a hypointense hue within the 3-mm zone (excluding the 1-mm zone). The foveal avascular zone (FAZ) was defined as an area within the innermost ring of the parafoveal capillaries if the FAZ border was clear. (**a–d**) Nonperfusion area 1 (NPA1) of the DCP was defined as the FAZ plus the nonperfusion area in the 1-mm zone. The NPA3s of (**a**) the SCP and (**b**) the DCP in recurrence cases were delineated as the yellow area. The NPA1s of (**a**) the SCP and (**b**) the DCP in a recurrence case were defined as the FAZ (gray area) plus microvascular nonperfusion area (blue area) in the 1-mm zone. (**c**) The NPA3 of the SCP and (**d**) DCP in a nonrecurrence case was also delineated as the yellow area. The NPA1s of (**c**) the SCP and (**d**) the DCP in this nonrecurrence case were also defined as the FAZ (gray area) plus microvascular nonperfusion area (blue area) in the 1-mm zone.

**Figure 3:**
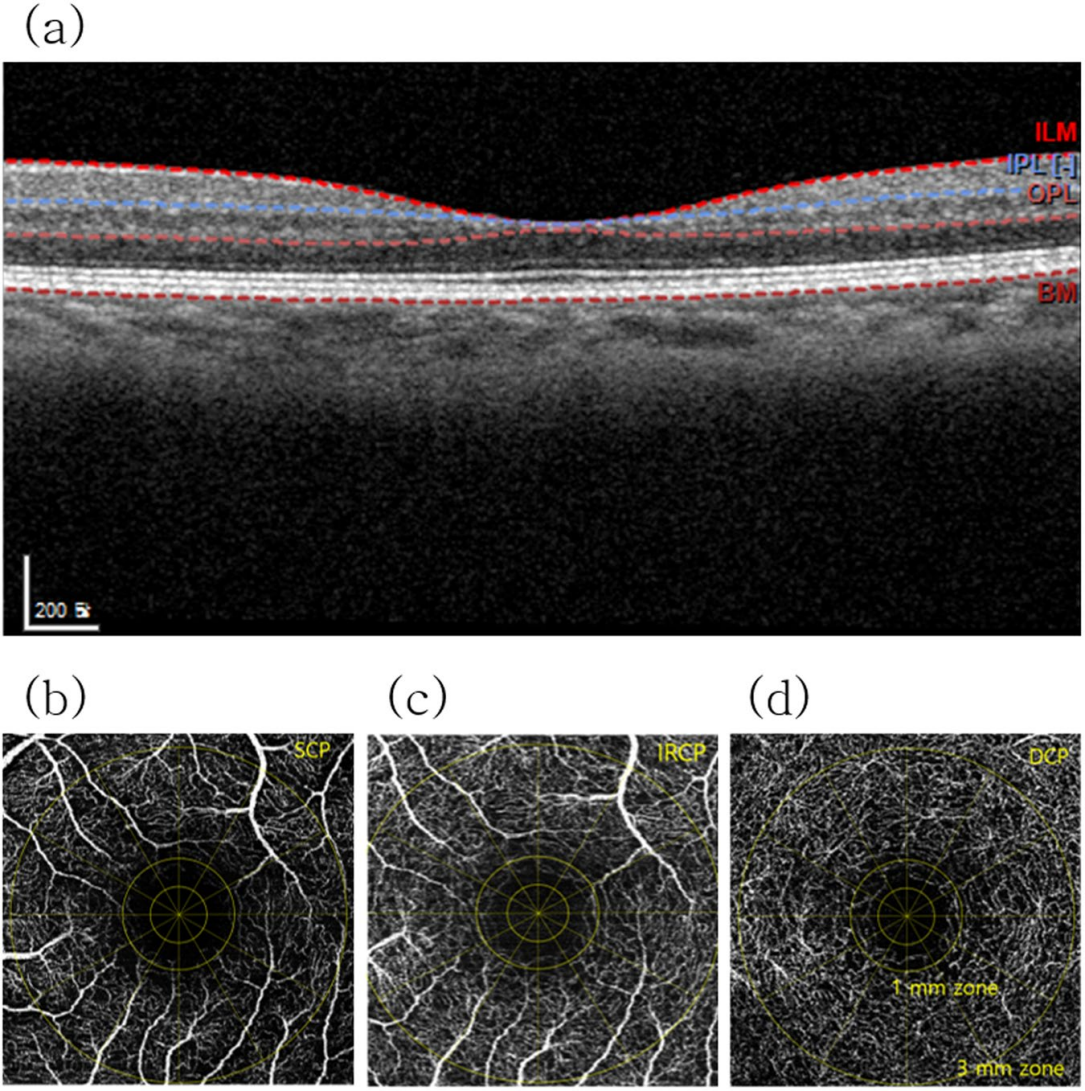
(**a**) OCT image and (**b**–**d**) OCTA images of the fellow eye in a 69-year-old male patient from the nonrecurrence group. OCTA image of (**d**) the DCP showing a more prominent FAZ than in (**b**) the SCP. Single-layered capillaries are shown to be adherent to the FAZ within (**c**) the inner retinal capillary plexus layer (IRCP) and (**d**) the DCP but not (**b**) the SCP.

Using the default automated segmentation boundaries in the built-in software, separate flow maps of the SCP and DCP were obtained for each eye. The location of the segmentation line between the SCP and DCP in our module was as indicated in Table 1. The fovea-centered OCTA images were manually overlaid with the ETDRS circle. The foveal avascular zone (FAZ) was defined as a lesion devoid of retinal vessels within the boundary of the innermost the parafoveal. In our BRVO cases, some cases were an intact oval-shaped FAZ, but the others was not with capillary dropouts and anastomotic arcade disruptions (Fig. 1)^31^. The NPA as identified in OCTA images was defined as the all dropout area of the retinal capillary beds with any dark or gray areas^32^, including the FAZ (Figs 1, 2). The two-layered capillaries at the border of the avascular zone ultimately join into a single-layered ring^20^, and the capillary plexus in the fovea was readily detected in the DCP using the Spectralis OCT device (Fig. 3). NPAs in the 1-mm zone (NPA1, including the FAZ) were measured at the CP by drawing the area contour point by point^33^ using ImageJ software (1.51J8 version, National Institutes of Health, Bethesda, MD, USA); NPAs in the 1 mm to 3 mm (NPA3) zone (excluding the 1-mm zone) were measured within both the SCP and DCP by the same method (Fig. 2)^31,34^. Measurement of the NPAs in the SCP and DCP was performed by two independent retina specialists (K.E.C., S.W.K.) who were both blinded to patient information. Other microvascular abnormalities (capillary telangiectasias, collateral vessels (venovenous drainage), and microaneurysms) were also compared between those with and without ME recurrence^27^, and OCT images before treatment were compared with OCTA images after ME resolution in all cases.

**Table 1:** OCTA Instruments.

	Spectralis (Heidelberg)	AngioPlex (Zeiss)	DRI (Topcon)	RTVue XR Avanti (Optovue)
Type Wavelength	Spectral domain 870 nm	Spectral domain 840 nm	Swept-source 1050 nm	Spectral domain 840 nm
OCTA algorithm	FS-ADA	OMAG	OCTARA	SSADA
A-scan rate	85 KHz	68 KHz	100 KHz	70 KHz
Repeat B-scan	4–7	4	4	2
Eye track	TruTrack	FastTrac	SMARTtrack	VTRAC
Segmentation boundary for the SCP	ILM and outer boundary of the IPL	ILM and approximation of the IPL (0.7*thickness between the ILM and the OPL from the ILM)	2.6 µm beneath the ILM and 15.6 µm beneath the interface of the IPL/INL	3 µm below the ILM and 15 µm below the inner boundary of the IPL
Segmentation boundary for the DCP	Outer boundary of the IPL and outer boundary of the OPL	Approximation of IPL (0.7*thickness between the ILM and the OPL from the ILM) and approximation of the OPL (110 μm from the RPE boundary)	15.6 µm beneath the interface of the IPL/INL and 70.2 µm beneath the IPL/INL	15 µm below the inner border of the IPL and 70 µm below the inner border of the IPL (≈outer border of OPL)

DCP = deep capillary plexus; FS-ADA = full-spectrum amplitude decorrelation algorithm; ILM = inner limiting membrane; IPL = inner plexiform layer; INL = inner nuclear layer; OCTA = optical coherence tomography angiography; OCTARA = OCTA ratio analyses; OMAG = OCT-microangiography complex algorithm; OPL = outer plexiform layer; SCP = superficial capillary plexus; SSADA = split spectrum amplitude decorrelation algorithm.

### Statistical analysis

Continuous variables are presented as mean ± SD and were analyzed using the Mann-Whitney test or Spearman correlation analysis. The other factors were compared between those with and without ME recurrence using the Pearson chi-square test, Fisher’s exact test, or Wilcoxon signed rank test, as appropriate. Multivariate logistic analyses using the significant parameters identified in the univariate analyses as the independent variables were performed to identify factors associated with ME recurrence. Statistical analyses were performed using SPSS version 21.0.0.0 (SPSS, Chicago, IL, USA) or MedCalc V.12.1.3.0 (MedCalc Software bvba, Ostend, Belgium). All statistics were two-tailed, and p values of <0.05 were considered statistically significant. The interclass correlation coefficient for each NPA measurement was calculated, and those mean values were used for the statistical analyses.

## Results

### Patient characteristics

A total of 40 eyes were enrolled in this study. ME did not recur within six months after the initial intravitreal anti-VEGF injections (one or two) in 15 of the 40 eyes (37.5%), whereas in 25 of 40 eyes (62.5%) it did recur. 14 of the 40 patients were male, and the other 26 patients were female. The mean age of the patients was 62.15 ± 12.66 years. Mean disease duration was 1.09 ± 1.02 months, and mean follow-up duration was 12.33 ± 5.99 months. The mean number of injections before recurrence within 6 months after ME resolution was 1.38 ± 0.49 (25 patients received one injection, and 15 patients received two injections). At the initial visit, BCVA (logMAR) was 0.58 ± 0.38, and at the last visit, it was 0.25 ± 0.24. There was no significant difference in the initial BCVA assessment between eyes with ME recurrence and those without ME recurrence (log MAR: 0.69 ± 0.44 and 0.49 ± 0.47, respectively; p = 0.376). Other characteristics of patients were also not statistically different between two groups (Table 2).

**Table 2:** Patient characteristics.

	Nonrecurrence (n = 15)	Recurrence (n = 25)	p value
Age (years)	57.60 ± 11.91	64.88 ± 12.54	0.083^a^
Sex (male/female)	7/8	7/18	0.231^b^
HTN (Y/N)	12/3	17/8	0.486^c^
DM (Y/N)	15/0	24/1	1.000^c^
Laterality (OD/OS)	6/9	7/18	0.433^b^
Lens (phakia/pseudophakia)	14/1	20/5	0.381^c^
Number of injections (one/two)	8/7	17/8*	0.354^b^
Initial BCVA (log MAR)	0.69 ± 0.44	0.49 ± 0.47	0.376^a^
1-mo BCVA	0.32 ± 0.29	0.40 ± 0.32	0.422^a^
3-mo BCVA	0.18 ± 0.29	0.29 ± 0.27	0.267^a^
Symptom duration (mo)	1.26 ± 1.13	0.99 ± 0.95	0.679^a^
PVD (Y/N)	12/3	16/9	0.477^c^

BCVA = best-corrected visual acuity; DM = diabetes mellitus; HTN = hypertension; mo = month; PVD = posterior vitreous detachment.

*Number of injections in the recurrence group was the number of injections before recurrence within 6 months after ME resolution following treatments.

^a^Mann-Whitney U test.

^b^Pearson chi-square test.

^c^Fisher’s exact test.

### OCT and OCTA findings

Prior to intravitreal anti-VEGF injection, the CRT was 477.40 ± 130.50 μm in the nonrecurrence group and 595.76 ± 167.81 μm in the recurrence group (p = 0.022). One month after the initial intravitreal anti-VEGF injection, the CRT was 303.22 ± 89.63 μm in the nonrecurrence group and 273.20 ± 56.79 μm in the recurrence group (p = 0.182). Three months after the initial intravitreal anti-VEGF injection, the CRT was 249.73 ± 28.96 μm in the nonrecurrence group and 377.24 ± 172.81 μm in the recurrence group (p = 0.028). There was no case of only serous detachment type without cyst, and the type of ME before anti-VEGF injection did not differ significantly between the two groups (p = 0.495).

The interclass correlation coefficients for the NPA measurements were >0.95. In each individual, there was no significant difference between the mean NPA3 of the DCP and that of the SCP (1.415 ± 0.708 mm^2^, 1.489 ± 0.797 mm^2^, respectively, p = 0.137 by Wilcoxon signed rank test). The mean NPA1 of the SCP was 0.441 ± 0.129 mm^2^ in the nonrecurrence group and 0.610 ± 0.176 mm^2^ in the recurrence group (p = 0.002). The mean NPA1 of the DCP was 0.473 ± 0.128 mm^2^ in the nonrecurrence group and 0.629 ± 0.169 mm^2^ in the recurrence group (p = 0.004). The mean NPA3 of the SCP was 1.005 ± 0.729 mm^2^ in the nonrecurrence group and 1.779 ± 0.701 mm^2^ in the recurrence group (p = 0.002). The mean NPA3 of the DCP was 1.021 ± 0.648 mm^2^ in the nonrecurrence group and 1.652 ± 0.643 mm^2^ in the recurrence group (p = 0.008). Microvascular aneurysms and telangiectasia were detected more in the DCP than in the SCP. None of the microvascular abnormalities differed significantly between the recurrence group and the nonrecurrence group (Table 3).

**Table 3:** Optical coherence tomography and optical coherence tomography angiography findings of the study patients.

	Nonrecurrence (n = 15)	Recurrence (n = 25)	p value
Initial macular edema type			0.277^a^
1. Cystoid	4	8	
2. Serous + cystoid	7	15	
3. Sponge-like	4	2	
Initial CRT (μm)	477.40 ± 130.50	595.76 ± 167.81	0.022^b^
One-month CRT (μm)	303.22 ± 89.63	273.20 ± 56.79	0.182^b^
Three-month CRT (μm)	249.73 ± 28.96	377.24 ± 172.81	0.028^b^
Microvascular abnormality	Y/N	Y/N	
Telangiectasias in SCP	10/5	10/15	0.102^c^
Collateral vessels in SCP	3/12	10/15	0.298^a^
Aneurysms in SCP	11/4	18/7	1.000^a^
Telangiectasias in DCP	15/0	25/0	
Collateral vessels in DCP	1/14	2/23	1.000^a^
Aneurysms in DCP	13/2	23/2	0.622^a^
NPA1 of SCP (mm^2^) Mean	0.441 ± 0.129	0.610 ± 0.176	0.002^b^
Examiner 1	0.442 ± 0.132	0.609 ± 0.173	0.003^b^
Examiner 2	0.439 ± 0.127	0.611 ± 0.181	0.003^b^
NPA3 of SCP (mm^2^) Mean	1.005 ± 0.729	1.779 ± 0.701	0.002^b^
Examiner 1	1.008 ± 0.729	1.768 ± 0.715	0.005^b^
Examiner 2	1.003 ± 0.715	1.789 ± 0.698	0.003^b^
NPA1 of DCP (mm^2^) Mean	0.473 ± 0.128	0.629 ± 0.169	0.004^b^
Examiner 1	0.476 ± 0.128	0.625 ± 0.174	0.006^b^
Examiner 2	0.469 ± 0.129	0.638 ± 0.169	0.001^b^
NPA3 of DCP (mm^2^) Mean	1.021 ± 0.648	1.652 ± 0.643	0.008^b^
Examiner 1	1.026 ± 0.645	1.645 ± 0.654	0.012^b^
Examiner 2	1.016 ± 0.647	1.659 ± 0.629	0.007^b^

CRT = central retinal thickness; DCP = deep capillary plexus; NPA1 = nonperfusion area in the 1-mm zone; NPA3 = nonperfusion area in the 1- to 3-mm zone; SCP = superficial capillary plexus.

^a^Fisher’s exact test.

^b^Mann–Whitney U test.

^c^Pearson chi-square test.

In the analysis of all cases, there was no significant correlation between ME type on the OCT images before treatment and any of the vascular abnormalities on the OCTA images after ME resolution. In addition, there was no significant correlation between each vascular abnormality after ME resolution and the CRT at each follow-up, as determined by the Mann-Whitney U test. The only significant correlation was between the NPA3 of the DCP in the OCTA images after ME resolution and the initial CRT in the OCT images (Rho = 0.353, p = 0.026 by Spearman correlation analysis).

### Univariate and multivariate regression analysis

The initial CRT (odds ratio [OR]: 1.005; p = 0.034), three-month CRT (OR:1.014; p = 0.031), mean NPA1 of the SCP (OR: 766.500; p = 0.002), mean NPA1 of the DCP (OR: 555.324; p = 0.009), mean NPA3 of the DCP (OR: 5.022; p = 0.012), and mean NPA3 of the SCP (OR: 4.376; p = 0.006) differed significantly between the recurrence and nonrecurrence groups. The multivariate logistic regression analysis of the initial CRT, three-month CRT, mean NPA1 of the SCP, mean NPA1 of the DCP, mean NPA3 of the SCP, and mean NPA3 of the DCP showed that only the mean NPA1 of the DCP (OR: 344.718; p = 0.029) and the mean NPA3 of the SCP (OR: 4.072; p = 0.018) were significantly associated with ME recurrence (Table 4). Receiver operating characteristic (ROC) curve analyses indicated that both an NPA1 of the DCP of >0.46 mm^2^ (area under the curve [AUC]: 0.768, sensitivity: 80%; specificity: 60%) and an NPA3 of the SCP of >1.1 mm^2^ (AUC: 0.784, sensitivity: 84%; specificity: 60%) were strongly associated with ME recurrence.

**Table 4:** Binary logistic regression analysis of recurrent macular edema secondary to branch retinal vein occlusion.

	Univariate binary logistic regression analysis		Multivariate binary logistic regression analysis	
	OR	p value	OR	p value
Initial CRT (μm)	1.005	0.034		0.073
Three-month CRT (μm)	1.014	0.031		0.083
Mean NPA1 of SCP (mm^2^)	766.500	0.008		0.804
Mean NPA3 of SCP (mm^2^)	4.376	0.006	4.072	0.018
Mean NPA1 of DCP (mm^2^)	555.324	0.009	344.718	0.029
Mean NPA3 of DCP (mm^2^)	5.022	0.012		0.564

CRT = central retinal thickness; DCP = deep capillary plexus; NPA1 = nonperfusion area in the 1-mm zone; NPA3 = non-perfusion area in the 1–3-mm zone; OR = odd ratio; SCP = superficial capillary plexus.

## Discussion

In the present study, we focused on the relationship between each NPA in the OCTA images and the recurrence of ME after intravitreal anti-VEGF injection in eyes with BRVO. Other possible risk factors such as microaneurysms in OCTA images did not differ significantly between the ME recurrence and nonrecurrence groups. The recurrence rate of ME within six months of the initial intravitreal anti-VEGF injection was 62.5% (25 of 40 eyes). We were unable to adequately evaluate the FAZ in the SCP using the Spectralis OCT device (Heidelberg) (Fig. 1). Single-layered vessels adherent to the FAZ were identified in our OCTA machine by comparing those images with images of the corresponding inner retinal capillary plexus layer (range: internal limiting membrane to basement membrane) (Fig. 3). In all cases, the DCP presented as a single layer of vessels near the FAZ, but the SCP in 6 cases (15%) presented as a partially intact single layer of vessels.

ME recurrence was correlated with the extent of the NPA1 (including the FAZ) of the DCP, NPA1 of the SCP, NPA3 of the DCP, NPA3 of the SCP, the initial CRT, and three-month CRT in both the Mann-Whitney test and univariate logistic regression analyses. In the multivariate logistic regression analysis, the extent of the NPA3 in the SCP and the NPA1 in the DCP were significantly associated with ME recurrence in BRVO patients. In our study, the extent of the NPA1 (including the FAZ) appeared to be consistent with the central retinal nonperfusion status, and the extent of the NPA3 was consistent with the parafoveal nonperfusion status. However, central hypoperfusion and parafoveal hypoperfusion were prominent in both the SCP and DCP in all cases, and there was no significant difference between the SCP and DCP. The NPA3s of the SCP and DCP were strongly correlated (Rho = 0.764, p < 0.001), and the NPA1s of the SCP and DCP were also strongly correlated (Rho = 0.876, p < 0.001).

Previous FA studies have demonstrated a relationship between ME and retinal nonperfusion in eyes with BRVO^35,36^. In addition, ME recurrence after intravitreal anti-VEGF injection in eyes with BRVO is associated with the initial extent of macular capillary nonperfusion as assessed by FA and foveal thickness as measured by OCT^16^. Recently, several OCTA studies have also suggested that, although both the superficial and deep vascular networks are severely affected in BRVO, microvascular abnormalities, including capillary nonperfusion, are more common in the DCP than in the SCP^27,28,32,37,38^. In previous OCTA studies, ME caused by retinal vein occlusion has been shown to occur in association with an absence of blood flow in the DCP or the difference in retinal capillary loss between the SCP and DCP^39,40^. On the other hand, one study showed that retinal sensitivity over the NPAs on both the SCP and DCP decreased, though the decrease was larger in the SCP^41^. Furthermore, at both CRVO and BRVO, changes in vascular density and complexity in the SCP were more severe than in the DCP^42^.

**Figure 4:**
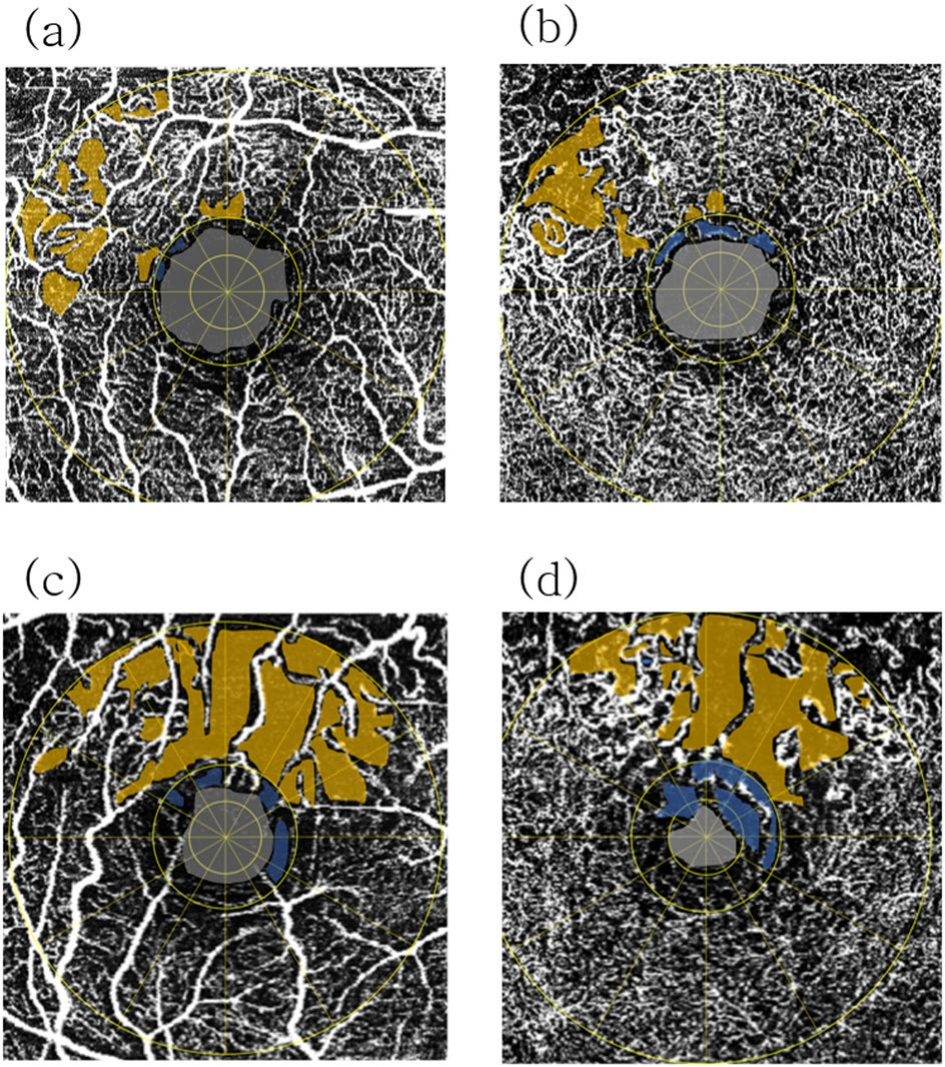
Schematic NPA images in (**a**,**b**) a 56-year-old female from the recurrence group and (**c**,**d**) a 57-year-old female from the nonrecurrence group. (**a**) The NPA3 (yellow area) and NPA1 (gray area plus blue area) of the SCP in this recurrence case were 0.414 mm^2^ and 0.564 mm^2^, respectively. (**b**) The NPA3 (yellow area) and NPA1 (gray area plus blue area) of the DCP in this recurrence case were 0.571 mm^2^ and 0.531 mm^2^, respectively. (**c**) The NPA3 (yellow area) and NPA1 (gray area plus blue area) of the SCP in this nonrecurrence case were 1.585 mm^2^ and 0.359 mm^2^, respectively. (**d**) The NPA3 (yellow area) and NPA1 (gray area plus blue area) of the DCP in this nonrecurrence case were 1.435 mm^2^ and 0.281 mm^2^, respectively.

The central retinal NPA (NPA1, including the FAZ) was also a prominent factor correlated with ME recurrence in our BRVO cases. Interestingly, some ME cases with large parafoveal NPAs (NPA3) did not recur, whereas some cases with moderate parafoveal NPAs did recur. Notably, in the nonrecurrence group, cases with large parafoveal NPAs had small central retinal NPAs, whereas in the recurrence group, cases with moderate parafoveal NPAs had large central retinal NPAs, which were found close in size of the 1-mm zone (Fig. 4).

After treatment of retinal vein occlusion with anti-VEGF agents, both short-term and long-term studies have found no significant differences in the FAZ area between CRVO and BRVO patients^43,44^. Adhi *et al*. showed that the FAZ area was larger in eyes with RVO than in their fellow eyes and controls, and in the fellow eyes of RVO patients when compared to controls^32^, Rispoli *et al*. reported similar results^37^. Yoo *et al*. showed that the degree of macular ischemia (not including the FAZ) as assessed by FA was associated with ME recurrence after intravitreal anti-VEGF injection^16^.

Our study had some limitations. First, all data were gathered retrospectively from one clinic using a particular kind of OCTA device. There are quite prominent differences among different OCTA devices with respect to tracing the superficial and deep capillary networks within the macular 1-mm zone (Table 1). Different OCT devices use different techniques to differentiate blood vessels by depicting changes in the OCT signal induced by moving blood cells^45^. In our OCTA study using the Spectralis (Heidelberg) imaging system, the FAZ size in the SCP was larger than in the DCP, and vascular complexes adherent to the FAZ were readily detected only within the DCP. Previous studies have generally presented OCTA analyses in BRVO using other OCA devices, such the RTVue XR 100 (Optovue)^9,25,27–32,34,37–40,42,44,46–50^, and AngioPlex (Zeiss)^33^, and found that the FAZ of the SCP was smaller than the FAZ of the DCP. The differences between the settings used to identify the interface between the SCP and DCP provided by each OCTA software package could affect the distinction of the en-face image (Table 1). Furthermore, although we tried to minimize the effect of artifacts by excluding poor-quality scans and OCTA images with significant artefacts^46^, image quality could have affected the vascular density measurements.

Second, we did not assess preoperative OCTA information, which is important to consider. Although some studies showed no significant differences in the OCTA findings of BRVO with ME after short-term anti-VEGF treatment^40,43,44^, other studies showed that the foveal microstructure could be altered following anti-VEGF or steroid treatment^50,51^. The obstructed capillaries might not be completely re-perfused even after successful treatment of the ME in cases where deep capillary ischemia persists^32,52^. Nevertheless, we did not routinely evaluate OCTA images before the intravitreal injections, because retinal hemorrhage following BRVO diminished image quality. Valerie Mane *et al*. showed that cystoid spaces were located within capillary dropout areas in chronic diabetic macular edema (DME). The en-face of OCTA could detect cyst lesions in the DME cases^52^, but also in the BRVO cases^29,53^. However, detecting cyctic lesion in BRVO cases is more difficult than in DME cases because intraretinal hemorrhage following BRVO obscured the OCTA signal from the retinal layers involved in and beneath the hemorrhage^53,54^. In those situations, detecting the flow signal in OCT and OCTA images before treatment was also difficult. Fortunately, Kia Tarassoly *et al*. showed that vessel density and FAZ did not differ significantly between groups with and without cystoid change^55^. The impact of microvascular abnormality findings in the initial OCTA images on the risk of recurrence of BRVO-related ME remain to be clarified.

Third, the number of injections differed between the recurrence and nonrecurrence groups, although that difference was not statistically significant (p = 0.376 by Mann-Whitney U test, 0.898 by multivariate regression analysis). The treatment protocol of our study varies from the established protocol of 3 monthly anti-VEGF injections followed by PRN dosing. A further study with protocol of 3 monthly anti-VEGF injections for both groups could support our results.

In conclusion, our study demonstrates the utility of OCTA in quantifying vascular abnormalities, especially vascular nonperfusion in each of two capillary plexuses in BRVO eyes with ME recurrence. We found that nonperfusion status was strongly correlated with ME recurrence, especially in central foveal and parafoveal nonperfusion. Given its noninvasive nature and detailed depth-resolved vascular mapping ability, OCTA could become a preferred tool for the diagnosis and longitudinal evaluation of BRVO and other vascular occlusive diseases. Future studies comparing NPAs among various OCTA images and evaluating retinal vascular changes over time in response to therapy might be needed.

## Data Availability

The datasets generated and analyzed during the current study are available from the corresponding author upon reasonable request.

## References

[1] McIntosh RL (2010). Natural history of central retinal vein occlusion: an evidence-based systematic review. Ophthalmology.

[2] Hayreh SS, Zimmerman MB (2014). Branch retinal vein occlusion: natural history of visual outcome. JAMA Ophthalmol.

[3] Hayreh SS, Zimmerman MB (2015). Fundus changes in branch retinal vein occlusion. Retina.

[4] Yau JW, Lee P, Wong TY, Best J, Jenkins A (2008). Retinal vein occlusion: an approach to diagnosis, systemic risk factors and management. Intern Med J.

[5] Jaulim A, Ahmed B, Khanam T, Chatziralli IP (2013). Branch retinal vein occlusion: epidemiology, pathogenesis, risk factors, clinical features, diagnosis, and complications. An update of the literature. Retina.

[6] Argon laser photocoagulation for macular edema in branch vein occlusion (1984). The Branch Vein Occlusion Study Group. Am J Ophthalmol.

[7] Argon laser scatter photocoagulation for prevention of neovascularization and vitreous hemorrhage in branch vein occlusion (1986). A randomized clinical trial. Branch Vein Occlusion Study Group. Arch Ophthalmol.

[8] Finkelstein D (1992). Ischemic macular edema. Recognition and favorable natural history in branch vein occlusion. Arch Ophthalmol.

[9] Winegarner A (2018). Retinal Microvasculature and Visual Acuity After Intravitreal Aflibercept in Eyes with Central Retinal Vein Occlusion: An Optical Coherence Tomography Angiography Study. Retina.

[10] Aiello LP (1994). Vascular endothelial growth factor in ocular fluid of patients with diabetic retinopathy and other retinal disorders. N Engl J Med.

[11] Noma H (2005). Pathogenesis of macular edema with branch retinal vein occlusion and intraocular levels of vascular endothelial growth factor and interleukin-6. Am J Ophthalmol.

[12] Brown DM (2011). Sustained benefits from ranibizumab for macular edema following branch retinal vein occlusion: 12-month outcomes of a phase III study. Ophthalmology.

[13] Clark WL (2016). Intravitreal Aflibercept for Macular Edema Following Branch Retinal Vein Occlusion: 52-Week Results of the VIBRANT Study. Ophthalmology.

[14] Heier JS (2012). Ranibizumab for macular edema due to retinal vein occlusions: long-term follow-up in the HORIZON trial. Ophthalmology.

[15] Jaissle GB (2011). Predictive factors for functional improvement after intravitreal bevacizumab therapy for macular edema due to branch retinal vein occlusion. Graefes Arch Clin Exp Ophthalmol.

[16] Yoo JH, Ahn J, Oh J, Cha J, Kim SW (2017). Risk factors of recurrence of macular oedema associated with branch retinal vein occlusion after intravitreal bevacizumab injection. Br J Ophthalmol.

[17] Parodi MB, Visintin F, Della Rupe P, Ravalico G (1995). Foveal avascular zone in macular branch retinal vein occlusion. Int Ophthalmol.

[18] Park JJ, Soetikno BT, Fawzi AA (2016). Characterization of the Middle Capillary Plexus Using Optical Coherence Tomography Angiography in Healthy and Diabetic Eyes. Retina.

[19] Genevois O (2004). Microvascular remodeling after occlusion-recanalization of a branch retinal vein in rats. Invest Ophthalmol Vis Sci.

[20] Snodderly DM, Weinhaus RS, Choi JC (1992). Neural-vascular relationships in central retina of macaque monkeys (Macaca fascicularis). J Neurosci.

[21] Paques M (2003). Structural and hemodynamic analysis of the mouse retinal microcirculation. Invest Ophthalmol Vis Sci.

[22] Foreman DM (1996). Three dimensional analysis of the retinal vasculature using immunofluorescent staining and confocal laser scanning microscopy. Br J Ophthalmol.

[23] Foulds WS, Kek WK, Luu CD, Song IC, Kaur C (2010). A porcine model of selective retinal capillary closure induced by embolization with fluorescent microspheres. Invest Ophthalmol Vis Sci.

[24] Freund KB (2018). Association of Optical Coherence Tomography Angiography of Collaterals in Retinal Vein Occlusion With Major Venous Outflow Through the Deep Vascular Complex. JAMA Ophthalmol.

[25] Savastano MC, Lumbroso B, Rispoli M (2015). *In Vivo* Characterization of Retinal Vascularization Morphology Using Optical Coherence Tomography Angiography. Retina.

[26] de Carlo TE, Romano A, Waheed NK, Duker JS (2015). A review of optical coherence tomography angiography (OCTA). Int J Retina Vitreous.

[27] Suzuki N (2016). Microvascular Abnormalities on Optical Coherence Tomography Angiography in Macular Edema Associated With Branch Retinal Vein Occlusion. American journal of ophthalmology.

[28] Coscas F (2016). Optical Coherence Tomography Angiography in Retinal Vein Occlusion: Evaluation of Superficial and Deep Capillary Plexa. Am J Ophthalmol.

[29] Samara WA (2016). Quantitative Optical Coherence Tomography Angiography Features and Visual Function in Eyes With Branch Retinal Vein Occlusion. Am J Ophthalmol.

[30] Shiono A (2018). Optical coherence tomography findings as a predictor of clinical course in patients with branch retinal vein occlusion treated with ranibizumab. PLoS One.

[31] Seknazi D (2018). Optical Coherence Tomography Angiography in Retinal Vein Occlusion: Correlations Between Macular Vascular Density, Visual Acuity, and Peripheral Nonperfusion Area on Fluorescein Angiography. Retina.

[32] Adhi M (2016). Retinal Capillary Network and Foveal Avascular Zone in Eyes with Vein Occlusion and Fellow Eyes Analyzed With Optical Coherence Tomography Angiography. Invest Ophthalmol Vis Sci.

[33] Durbin MK (2017). Quantification of Retinal Microvascular Density in Optical Coherence Tomographic Angiography Images in Diabetic Retinopathy. JAMA Ophthalmol.

[34] Kadomoto S (2018). Evaluation Of Macular Ischemia in Eyes With Branch Retinal Vein Occlusion: An Optical Coherence Tomography Angiography Study. Retina.

[35] Prasad PS, Oliver SC, Coffee RE, Hubschman JP, Schwartz SD (2010). Ultra wide-field angiographic characteristics of branch retinal and hemicentral retinal vein occlusion. Ophthalmology.

[36] Noma H (2006). Intravitreal levels of vascular endothelial growth factor and interleukin-6 are correlated with macular edema in branch retinal vein occlusion. Graefes Arch Clin Exp Ophthalmol.

[37] Rispoli M, Savastano MC, Lumbroso B (2015). Capillary Network Anomalies in Branch Retinal Vein Occlusion on Optical Coherence Tomography Angiography. Retina.

[38] Wakabayashi T (2017). Retinal Microvasculature and Visual Acuity in Eyes With Branch Retinal Vein Occlusion: Imaging Analysis by Optical Coherence Tomography Angiography. Invest Ophthalmol Vis Sci.

[39] Tsuboi K, Ishida Y, Kamei M (2017). Gap in Capillary Perfusion on Optical Coherence Tomography Angiography Associated With Persistent Macular Edema in Branch Retinal Vein Occlusion. Invest Ophthalmol Vis Sci.

[40] Spaide RF (2016). Volume-Rendered Optical Coherence Tomography of Retinal Vein Occlusion Pilot Study. Am J Ophthalmol.

[41] Manabe S (2017). Association between Parafoveal Capillary Nonperfusion and Macular Function in Eyes with Branch Retinal Vein Occlusion. Retina.

[42] Koulisis N (2017). Quantitative microvascular analysis of retinal venous occlusions by spectral domain optical coherence tomography angiography. PLoS One.

[43] Farinha C (2015). Treatment of Retinal Vein Occlusion with Ranibizumab in Clinical Practice: Longer-Term Results and Predictive Factors of Functional Outcome. Ophthalmic Res.

[44] Ghasemi FK (2017). Optical Coherence Tomography Angiography Analysis of the Foveal Avascular Zone and Macular Vessel Density After Anti-VEGF Therapy in Eyes With Diabetic Macular Edema and Retinal Vein Occlusion. Invest Ophthalmol Vis Sci.

[45] Zhang A, Zhang Q, Chen CL, Wang RK (2015). Methods and algorithms for optical coherence tomography-based angiography: a review and comparison. J Biomed Opt.

[46] Spaide RF, Fujimoto JG, Waheed NK (2015). Image Artifacts in Optical Coherence Tomography Angiography. Retina.

[47] Spaide RF, Klancnik JM, Cooney MJ (2015). Retinal vascular layers imaged by fluorescein angiography and optical coherence tomography angiography. JAMA Ophthalmol.

[48] Lee J, Moon BG, Cho AR, Yoon YH (2016). Optical Coherence Tomography Angiography of DME and Its Association with Anti-VEGF Treatment Response. Ophthalmology.

[49] Wons J (2016). Optical Coherence Tomography Angiography of the Foveal Avascular Zone in Retinal Vein Occlusion. Ophthalmologica.

[50] Mastropasqua R (2017). Optical coherence tomography angiography microvascular findings in macular edema due to central and branch retinal vein occlusions. Sci Rep.

[51] Feucht N (2013). Changes in the foveal microstructure after intravitreal bevacizumab application in patients with retinal vascular disease. Clin Ophthalmol.

[52] Mane V (2016). Correlation between Cystoid Spaces in Chronic Diabetic Macular Edema and Capillary Nonperfusion Detected by Optical Coherence Tomography Angiography. Retina.

[53] Kashani AH, Lee SY, Moshfeghi A, Durbin MK, Puliafito CA (2015). Optical Coherence Tomography Angiography of Retinal Venous Occlusion. Retina.

[54] Moult E (2014). Ultrahigh-speed swept-source OCT angiography in exudative AMD. *Ophthalmic Surg Lasers Imaging*. Retina.

[55] Tarassoly K, Miraftabi A, Soltan Sanjari M, Parvaresh MM (2018). The Relationship between Foveal Avascular Zone Area, Vessel Density, and Cystoid Changes in Diabetic Retinopathy: An Optical Coherence Tomography Angiography Study. Retina.

